# Optimising costs in reducing rate of falls in older people with the improvement of balance by means of vestibular rehabilitation (ReFOVeRe study): a randomized controlled trial comparing computerised dynamic posturography vs mobile vibrotactile posturography system

**DOI:** 10.1186/s12877-018-1019-5

**Published:** 2019-01-03

**Authors:** Andrés Soto-Varela, Pilar Gayoso-Diz, Ana Faraldo-García, Marcos Rossi-Izquierdo, Isabel Vaamonde-Sánchez-Andrade, María del-Río-Valeiras, Antonio Lirola-Delgado, Sofía Santos-Pérez

**Affiliations:** 1 Division of Neurotology, Department of Otorhinolaryngology, Complexo Hospitalario Universitario, Santiago de Compostela, Spain; 2 Department of Surgery and Medical-Surgical Specialities, University of Santiago de Compostela, Santiago de Compostela, Spain; 3 Clinical Epidemiology Unit, Hospital Clínico Universitario. Instituto de Investigación Sanitaria de Santiago (IDIS), Santiago de Compostela, Spain; 4 Department of Otorhinolaryngology, Complexo Hospitalario Universitario, Santiago de Compostela, Spain; 5 Department of Otorhinolaryngology, University Hospital Lucus Augusti, Lugo, Spain

**Keywords:** Vestibular rehabilitation, Falls in elderly, Computerized dynamic posturography, Mobile posturography, Chronic dizziness

## Abstract

**Background:**

Accidental falls, especially for the elderly, are a major health issue. Balance disorders are one of their main causes. Vestibular rehabilitation (VR) has proven to be useful in improving balance of elderly patients with instability. Its major handicap is probably its cost, which has prevented its generalisation. So, we have designed a clinical trial with posturographic VR, to assess the optimum number of sessions necessary for a substantial improvement and to compare computerised dynamic posturography (CDP) (visual feedback) and mobile posturography (vibrotactile feedback).

**Methods:**

*Design*: randomized controlled trial. It is an experimental study, single-center, open, randomized (balanced blocks of patients) in four branches in parallel, in 220 elderly patients with high risk of falls; follow-up period: twelve months. *Setting*: Department of Otorhinolaryngology of a tertiary referral hospital. *Participants*: people over 65 years, fulfilling two or more of the following requirements: a) at least one fall in the last twelve months. b) take at least 16 s or require some support in perform the “timed up and go” test. c) a percentage of average balance in the sensory organization test (SOT) of the CDP < 68%. d) at least one fall in any of the conditions in SOT-CDP. e) a score in Vertiguard’s gSBDT > 60%. *Intervention*: Four differents protocols of vestibular rehabilitation (randomization of the patients). *Main outcome measure*: The percentage of average balance in the SOT-CDP. *Secondary measures*: time and supports in the “timed up and go” test, scores of the CDP and Vertiguard, and rate of falls.

**Discussion:**

Posturographic VR has been proven to be useful for improving balance and reducing the number of falls among the aged. However, its elevated cost has limited its use. It is possible to implement two strategies that improve the cost-benefit of posturography. The first involves optimising the number of rehabilitation sessions; the second is based on the use of cheaper posturography systems.

**Trial registration:**

ClinicalTrials.gov identifier: NCT03034655. Registered on 25 January 2017.

## Background

Accidental falls, especially for the elderly, are a major health problem in Western society [1, 2]. Approximately one-third of those over 65 suffer at least one fall per year; among those over the age of 85, this rises to approximately half. Furthermore, those who fall will often fall regularly (several times per year).

The repercussions of these falls in terms of morbimortality are serious, particularly because of associated bone fractures. Especially grave are hip fractures. Even if bone fractures do not result from the fall, the psychological consequences of falls seriously affect the quality of life of those affected. People who have suffered a fall tend to fear that this will happen again, and thus to reduce their physical activity. This, in turn, increases the chances of new falls and increases levels of fear, in a vicious circle that often leads to isolation and increasing dependency on carers [3].

In addition to these clinical and psychological consequences, the economic repercussions of the problem are also serious. The direct healthcare expenditure of these falls is very high, and the indirect cost of the recovery process and the monitoring thereafter is also very significant; there is the cost of increased care, and of adapting the home to the person’s new needs (ramps for wheelchairs, lifts and/or elevating platforms, handles in bathrooms) as well as the expense of other mobility equipment (walking supports, crutches, walking frames, etc.).

Many factors contribute to the risk of falls [4], including problems in the central nervous system that affect coordination and motion, balance disorders of different origins (vestibular, locomotor, visual, etc.), and advanced age [5] and age-related sensorial impairments [6, 7]. Several strategies can minimise risk [8, 9]; it is important to identify those in gravest danger of falling, in order to implement the necessary precautionary measures and to prevent (or at least to slow down) the age-related causes of falls using training and rehabilitation.

Vestibular rehabilitation has proven to be useful in improving the balance of elderly patients with instability [10–12]. Several protocols for vestibular rehabilitation exist (home exercises, Tai-Chi [13], optokinetic stimulus, rotating chairs, dynamic posturography exercises, etc.). Dynamic computerized posturography has proven to be especially useful for elderly patients, because it not only improves the stabilisation of the centre of gravity (where sensorial stimuli are absent or altered), but also improves the stability levels of the patient (and thus reduces the risk of falling) [14, 15]. Its major handicap is probably its cost, which has prevented its dissemination and generalisation.

Two possible solutions are presented here, which may serve to reduce this cost. One of these is optimising and reducing the number of vestibular rehabilitation sessions. This strategy has already proven useful for patients whose lack of balance is caused by reasons other than age [16]. The other option is using cheaper posturography systems; for example, Vertiguard® (Vesticure GmbH, Germany), which allows for the analysis of the motion of the centre of gravity in the course of daily activities, in both static and walking patients, as well as vibrotactile stimulation feedback-assisted rehabilitation [17]. This system has proven to be effective with different pathologies [18, 19], but no extensive and systematic studies have yet been undertaken that compare it with dynamic posturography in the treatment of age-related balance deficits.

For this reason, we suggest that it would be fruitful to undertake a study of vestibular rehabilitation in unstable aged patients, which will permit a double assessment. This study will:

- Evaluate the optimum number of sessions necessary for a substantial improvement (five vs ten);
- Compare computerised dynamic posturography (visual feedback) and mobile posturography (vibrotactile feedback).

This study was funded by the project PI1500329, integrated into the Spanish State Plan for R + D + I and funded by the ISCIII-Subdirección general de Evaluación y Fomento de la Investigación and the Fondo Europeo de Desarrollo regional (FEDER).

## Methods and design

### Aim of the study

The primary objective is to evaluate the effectiveness of a mobile posturographic system with vibrotactile neurofeedback to improve balance in elderly patients. Each subject was assessed immediately after the rehabilitation.

The secondary objectives are:

- To verify that improvements in balance are maintained in the medium term (six to twelve months).
- To consider whether this improvement in balance results in a reduction in the number of falls suffered by the elderly.
- To assess whether the reduction in the number of vestibular rehabilitation sessions (five) leads to similar results (an improvement in balance and a reduction in the number of falls) to those obtained after ten sessions. Both rehabilitation systems are compared using computerised dynamic posturography and mobile posturographic with vibrotactile neurofeedback.

### Design

This study is the continuation of a previous clinical trial conducted by our research group [14], comparing three different rehabilitation strategies (dynamic posturography exercises, optokinetic stimuli and exercises at home) and a control group, to improve balance in elderly. The design of both studies is similar.

This is a randomized controlled trial. It is an experimental study, single-centre, open-label and randomized (balanced blocks of patients) in four parallel branches. It involves 220 elderly patients (over 65 years of age) who are at high risk of falling, and a 12-month follow-up period will be applied.

### Study duration

The study period is 36 months.

### Study subjects

**Study population**: people over 65 years of age.

**Target population**: people over 65 years at a high risk of falling, from the Santiago de Compostela area.

Subjects > 65 years of both genders, at a high risk of falling (according to criteria to be described), will be included. These people will be selected from a larger population categorised according to one of the following characteristics:

- Patients > 65 years who visit the Neurotology Unit with balance disorders, in whom causes other than advanced age have been ruled out.
- Patients with no subjective balance disorder, who have not been seen for this reason, who agree to undergo the examination protocol; for these patients, it must be verified that they meet the established fall risk criteria.

**Pre-screening visit 0**: This involves a complete balance examination and interview that enables us to select individuals with balance disorders who are at a high risk of falling and are thus candidates for inclusion in the study.

This visit includes the following:

- Questionnaires that measure disability caused by imbalance and risk of falling. Such questionnaires include:

- Direct questions about the number of falls over the last 12 months.
- Dizziness Handicap Inventory (DHI), validated in Spanish [20], and a shortened version of the Falls Efficacy Scale-International (FES-I) to assess fear of falling (Short FES-I) [21], which evaluates the fear of falling while performing seven everyday activities. An explanation of scores of these questionnaires has been previously published [14].

b) Modified “timed up and go” (TUG) test [22, 23]: the subject, seated, must stand up unaided, walk 3 m, turn around and sit down again. The time needed, the number of steps taken and the need for support are determined.
c) Computerised dynamic posturography (CDP) sensory organisation test (SOT) and limits of stability (LOS); we used the Neurocom® Smart Equitest platform. Performing of SOT and LOS has been previously published [14].
d) Studying the balance record using the mobile Vertiguard® system: 13 tasks are performed; their analysis represents the geriatric Standard Balance Deficit Test (gSBDT):

- Standing still (SS), eyes open, normal surface (NS).
- SS, eyes closed, NS.
- 8 steps in tandem, eyes open, NS.
- SS, eyes open, foam surface (FS).
- SS, eyes closed, FS.
- 8 steps in tandem, eyes open, FS.
- Walk 3 m, eyes open.
- Walk 3 m, eyes open, turning the head from side to side.
- Walk 3 m, eyes open, moving the head up and down.
- Walk 3 m, eyes closed.
- Walk over 4 barriers (height: 26 cm; distance between barriers: 1 m).
- Sit on a chair.
- Get up from a chair.

The subject’s eligibility will be verified upon completion of these tests. If he/she meets the inclusion criteria and none of the exclusion criteria apply, then the investigator will provide him/her with a detailed, systematic explanation of the study’s objectives and what participation involves, inviting him/her to join. The subject will be given the approved information sheet in order to obtain his/her written informed consent.

### Inclusion criteria

Persons at a high risk of falling shall meet at least two of the following requirements:

- They have fallen at least once over the last 12 months.
- They required more than 15 s to complete, or require support in, the TUG test.
- They obtained a mean CDP SOT balance score of < 68%.
- They have fallen at least once in the CDP SOT.
- They obtained a score in Vertiguard’s gSBDT of > 60%.

### Exclusion criteria

- Cognitive decline or reduced cultural level that prevents the patient from understanding the assessment, undertaking the vestibular rehabilitation exercises and providing informed consent.
- Organic conditions that prevent the patient from standing on two feet, which is necessary for the assessment of balance and performance of VR exercises.
- Balance disorders caused by conditions other than age (neurological, vestibular, etc.).
- Undergoing treatment which may have an effect on balance.

### Calculation of sample size

The sample size was estimated after consulting the results of previous studies conducted by the group [24] in which there was a 10-point change in the mean balance CDP SOT (baseline value: 64; post-rehabilitation value: 73; value after 12 months: 74). We considered that a security level (1-ά) of 95% and a type II (β) error likelihood of 0.2, 53 subjects are needed for each arm. The sample size will be 212 subjects; it will be increased to 220 (55 for each arm) in case of any loss to follow-up.

### Randomisation and intervention

#### Visit 1

After the first screening visit, the patients who provided consent will be included in the study and randomised to one of the following study arms:

A. **Intervention with dynamic computerized posturography exercises (ten sessions)**. The Smart Equitest program was used: 10 exercises per session, which were customized depending on each patient’s deficit (as observed in the earlier postural study), were undertaken. The exercises involve visual biofeedback together with sensitive, real-time monitoring of movement. For some exercises, patients must maintain their centre of gravity (COG) over the base of support, while for others the COG must be moved to a series of targets. In addition, the support surface and/or visual surround may also move in response to the patient’s own movement. The exercise difficulty was progressively increased throughout the rehabilitation sessions by increasing the LOS, the transition rate or the movement of the posturography platform. The duration of each session was approximately 15 min. The distribution of sessions was one per day and five per week (two weeks)
B. **Intervention with dynamic computerized posturography exercises (five sessions)**. Same as Group A, except for the number of sessions (5) and their distribution (one session on alternate days, for two weeks).
C. **Intervention with mobile posturography (Vertiguard) exercises (ten sessions)**. As many as six tasks were set, and the most prominent deviations from normative control values were included in the training programme. Individual feedback was stored in the device for each patient based on body sway analysis. Training was performed using the training function of Vertiguard-RT device. This neurofeedback system contains a battery-driven main unit which is fixed on a belt at the centre of the body mass (hip) and one vibration stimulator on the front, back, left and right side, respectively. Vibration stimulators are mounted on the same belt as the main unit. They were adjusted by sliding them over the belt to the correct position for the individual patient. The main unit continuously determines the Coriolis force during body movements (pitch and roll) using inbuilt gyroscopes and compares those values with individual pre-set thresholds for stimulator activation in specific directions.
  Training was performed daily under the supervision of a physician over 2 weeks (over 10 sessions and the weekend was excluded). A training session consisted of 5 repetitions of six selected training tasks as described above (each repetition lasted 20 s or until the movement was finished). The patient received a vibrotactile feedback signal during training for those directions when a body sway higher than pre-set thresholds was noted. Vibration was reinforced with increasing sway: the more it exceeded the pre-set values, the stronger the vibration was at the corresponding site.
  No vibrotactile feedback was applied if the patient’s sway was below pre-set thresholds. The exercise difficulty progressively increased throughout the rehabilitation sessions by pressing the sensitivity buttons (up/down) on the main unit. During this procedure, the individual pre-set thresholds were similarly decreased for all sway directions of the specific training conditions until the patient perceived a vibration while performing the training task.
D. **Intervention with mobile device Vertiguard exercises (five sessions)**. Same as Group C, except for the number of sessions (5) and their distribution (one session on alternate days, for two weeks).

Randomisation will be performed by C.H.U de Santiago Clinical Epidemiology and the Biostatistics Unit, following the same strategy than in our previous clinical trial [14].

### Variables

The study variables shall be as follows:

1. Date of birth.
2. Sex.
3. Weight (kg) and height (cm).
4. Number of falls in the 12 months before inclusion.
5. Hospitalisations due to falls over the previous 12 months.
6. Percentage of mean balance (average) as observed in computerized dynamic posturography (CDP) sensory organisation test (SOT) (PRIMARY ENDPOINT); this will be obtained by calculating the mean of the scores of the 18 records of each sensory organisation test (calculated by software).
7. DHI score: covered by patient.
8. Short FES-I score: covered by patient.
9. Duration (seconds) of TUG: measured by evaluator.
10. Number of steps in TUG: measured by evaluator.
11. Number of supports in TUG: measured by evaluator.
12. Mean value of CDP SOT condition 5: calculated by software.
13. Mean value of CDP SOT condition 6: calculated by software.
14. CDP SOT vestibular contribution value: (mean value of condition 5/mean value of condition 1) × 100.
15. Mean value of directional control of limits of stability in CDP: calculated by software.
16. Mean endpoint value and maximum travel point of limits of stability in CDP: calculated by software.
17. Vertiguard gSBDT value: calculated by software.
18. Number of falls over 12 months after completion of VR programme (we will give the patient a timetable to allow them to check each fall immediately after it happens, in order to avoid memory bias).
19. Hospitalisation resulting from those falls: date, diagnosis, duration of hospitalisation.

### Data collection and ethics

The balance study undertaken in the first visit (**baseline record**) is repeated, with all the tests (questionnaires, TUG test, CDP SOT, CDP limits of stability and Vertiguard record) three more times:

● Visit 2.- Immediately after completion of VR.

● Visit 3.- Six months after VR.

● Visit 4.- Twelve months after VR.

The data will be collected in a specially designed database. There will be periodic data quality controls. The PI will monitor the study and ensure that the data are authentic, accurate and complete, and that the subjects’ safety and rights are protected.

The protocol has been approved by the Independent Ethics Committee of Galicia (protocol 2014/411).

The study will be conducted according to ICH Good Clinical Practices (GCP), the Declaration of Helsinki and Law 14/2007, of 3 July, on Biomedical Research.

### Study timeline

**Fig. 1:**
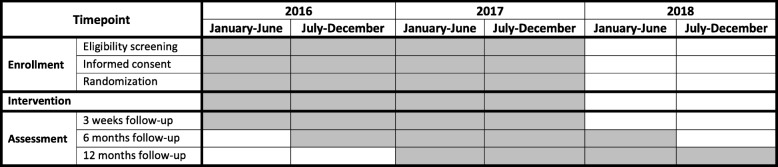
Standard protocol items: recommendations for interventional trials (SPIRIT) figure

The study is running from January 2016 to December 2019 (Fig. 1).

### Analytical plan and statistical measures

The patients’ demographic data and characteristics will be described globally and by the covariables of interest, as will the patients’ results.

The continuous results will be summarised as mean, median, standard deviation (D) and interquartile range (P25, P75). For the categorical results, we will present the number and percentage of patients in each category.

The statistical analyses will be based on the complete analysis group (CAG), which comprises all the subjects (those who granted informed consent and met all the inclusion and exclusion criteria, and were randomised to one of the study arms).

**The outcomes** of interest are: balance assessment (average balance score in CDP SOT and Vertiguard’s gSBDT score, with possible values 0–100 in both); assessment of instability perception scales (DHI, short FES-I) on visits 2 (after intervention), 3 (6 months after intervention) and 4 (12 months after intervention); number of falls at 12 months post-intervention.

**The covariables of interest** are:

- Baseline patient characteristics: they include the variables specified for visit 0.
- Associated risk factors: age, sex, weight (kg), height (cm), previous falls (yes/no).
- Adherence to intervention (investigator’s direct observation).

#### Bivariate analysis

We will provide the differences (95% CI) in the balance test assessments between baseline and end of intervention, and 6 months and 12 months post-intervention. These measures will be compared between groups by ANOVA or the Kruskall-Wallis test if normality is not assumed.

The Chi-squared test or Fisher’s exact test, depending on the conditions, will be used to analyse the number of falls occurring from 12 months prior to inclusion and 12 months post-intervention.

#### Multivariate analysis

In order to analyse the efficacy of the intervention in improving balance from the baseline, considering each subject’s repeated measures (visits 2, 3 and 4) and the covariables of interest, we will use mixed linear models (using Generalised Estimation Equations, GEE) when treatment is a fixed factor, the CDP SOT percentage mean balance at baseline and over time post-intervention is a random effect and a Gaussian distribution function is considered.

All the data analyses will be performed using R software, version 2.9.1 [25].

## Discussion

Reducing the number of falls among aged patients (and thus the related injuries and cost) is a goal that can be directly related to the ongoing 2020 European social programme (Healthcare, demographic change and well-being). One of the goals of this programme is to help aged patients stay active and healthy, by promoting active ageing, and a healthy and autonomous lifestyle, as well by helping aged patients to look after their own health. Aged population groups are especially exposed to cognitive impairment and are at risk of social exclusion. This has negative consequences on their independence and their quality of life, the independence of those who care for them and the sustainability of public healthcare systems. Europe faces a dwindling and ageing population. In 2050, the number of citizens aged over 65 will increase by 70%, and the number of those over 80 will grow by 170%.

Vestibular rehabilitation (and especially computerized dynamic posturography training) has been proven to be useful for improving balance and reducing the number of falls among the aged. However, the elevated cost of dynamic posturography has limited its use; the number of sessions currently prescribed (the average is approximately 10), limits the number of patients who can use this technique.

In order to increase efficiency and improve the cost-benefit function of vestibular rehabilitation as a mechanism to reduce the number of falls among aged patients, it is important to select particularly vulnerable groups. These groups can be established on the basis of several criteria, such as balance metrics [26, 27], age [28] and morphotypes [29]. We must prioritise these vulnerable groups.

It is also possible to implement two strategies that improve the cost-benefit of posturography. The first involves optimising the number of rehabilitation sessions. Although this number must be adjusted to the specific needs of each patient, the average is approximately 10. However, in certain patients who suffer from instability due to unilateral vestibular balance deficits, regardless of age, it has been proven that groups of five and of ten session programmes both have similar effects [16]. These data are, obviously, not directly extrapolate to elderly patients with instability, because in cases due to unilateral vestibular dysfunction, the deficit lies in only one labyrinth, and the deficit can be compensated with the remaining relevant sensorial systems (the other labyrinth, sight and the proprioceptive system) more easily. Age-related balance deficits, however, generally have multiple causes, and are a result of poorly managed sensory information. In any case, we think it is worth exploring this possibility because, if the results of five and ten rehabilitation session are similar, it would be possible to reduce the cost of vestibular rehabilitation by half, which would also increase the chances of patients undertaking all the rehabilitation treatment.

The second cost-reducing strategy is based on the use of cheaper posturography systems. One alternative to computerised dynamic posturography is the mobile posturography device Vertiguard® (Vesticure GmbH, Germany), which analyses the motions of the centre of gravity over the course of different daily activities, both static (standing with eyes open and closed in different postures) and walking (open and closed eyes, head in motion, navigating around obstacles) [17]. The device, which is placed on a belt on the patient’s waist, has four vibrators (front, back, left and right). These vibrators produce a vibrotactile stimulus which helps the patient maintain a stable centre of gravity over the course of different exercises, and thus the analysis can be undertaken while the patient is carrying out vestibular rehabilitation exercises. This device is considerably cheaper than dynamic posturography systems, and is of small size, which allows for ambulatory posturography vestibular rehabilitation. The efficiency of this technique has been demonstrated for the treatment of different pathologies [18, 19]. However, to date no extensive and systematic studies have been undertaken that compare it with dynamic posturography in the treatment of age-related balance deficits.

The main limitations of the study are the next:

- Despite being an open study, we do not believe that the VR and assessment tests being performed by the same person introduces substantial bias since the results are provided by software and not interpreted by an individual.
- When asking about the number of falls in the 12 months before inclusion, there could be memory bias. We will try to modulate this by requesting confirmation from a family member who lives with the patient. We will also check the information about falls over that period against the information contained in the patient’s electronic medical records (IANUS).

## Abbreviations

CDP: Computerised dynamic posturography
COG: Centre of gravity
DHI: Dizziness handicap inventory
FS: Foam surface
GCP: Good clinical practices
gSBDT: geriatric Standard Balance Deficit Test
LOS: Limits of stability
NS: Normal surface
Short FES-I: Shortened Version of the Falls Efficacy Scale-International
SOT: Sensory organization test
SS: Standing still
TUG: Timed up and go test
VR: Vestibular rehabilitation
